# A 5-Year Survival Prediction Model for Chronic Heart Failure Patients Induced by Coronary Heart Disease with Traditional Chinese Medicine Intervention

**DOI:** 10.1155/2021/4381256

**Published:** 2021-06-17

**Authors:** Hui Guan, Guo-Hua Dai, Wu-Lin Gao, Xue Zhao, Zhen-Hao Cai, Jia-Zhen Zhang, Jiu-Xiu Yao

**Affiliations:** ^1^First Clinical Medical College, Shandong University of Traditional Chinese Medicine, Jinan 250014, Shandong, China; ^2^Affiliated Hospital of Shandong University of Traditional Chinese Medicine, Jinan 250011, Shandong, China

## Abstract

**Objective:**

This study aimed to construct a 5-year survival prediction model of coronary heart disease (CHD) induced chronic heart failure (CHF), which is supported by the traditional Chinese medicine (TCM) factor, and to verify the model.

**Methods:**

Inpatients from January 1, 2012, to December 31, 2017, in seven hospitals in Shandong Province were studied. The random number table was used to randomly divide the seven hospitals into two groups (training set and verification set). In the training set, the least absolute shrinkage selection operator regression was first used to screen the independent variables. Logistic regression was then applied to construct a survival prediction model. The following nomogram visualizes the prediction model results. Finally, C-indices, calibration curves, and decision curves were used to discriminate and calibrate the established model and evaluate its practicability in the clinic. Bootstrap resampling and the verification set were used for internal and external verification, respectively.

**Results:**

A total of 424 eligible patients were included in the model construction and verification. In this 5-year survival prediction model of patients with CHF induced by CHD, eight independent predictors were included. The series of C-indices for the training set, bootstrap resamples, and verification set was 0.885, 0.867, and 0.835, respectively, demonstrating the credibility of our model. Additionally, the receiver operating characteristic curve, calibration curve, and clinical decision curve analysis of the training and verification sets showed that this 5-year survival prediction model was good in discrimination, calibration, and clinical practicability.

**Conclusion:**

This work highlights eight independent factors affecting 5-year mortality in patients with CHF induced by CHD after discharge and further helps reallocate medical resources rationally by precisely identifying high-risk groups. The constructed prediction model not only plays a credible role in prediction but also demonstrates TCM intervention as a protective factor for the 5-year death of patients with CHF induced by CHD, thereby advancing the use of TCM in CHF.

## 1. Introduction

Heart failure (HF) is a multi-incentive disease characterized by a structural or functional disorder of the myocardium, which results in decreased pumping power or filling function, a common end of most cardiovascular diseases. Chronic heart failure (CHF) is persistent HF that can stabilize, worsen, or decompensate. As the proportion of ageing society increases globally, the capacity for diagnosis and treatment of cardiovascular diseases, especially acute myocardial infarction, has greatly improved. Followed by the prevalence and incidence of patients with CHF exhibit an upward trend [1]. According to surveys, the prevalence of CHF in developed countries is approximately 1−2%, and the annual incidence is approximately 0.5−1%. In China, the prevalence of CHF is about 1.3%. That is, about 13.7 million people suffer from CHF. Although evidence-based treatment has reduced the mortality of CHF, the mortality in severe cases still approaches 50%, even worse than the prognosis of the common cancers [2]. Therefore, seeking an effective approach to improving the survival rate of CHF has become an urgent medical challenge worldwide [3].

Since accurate assessment of prognosis is a prerequisite for later rational treatment strategies, it is of great significance to develop credible models to select prognostic factors. To date, a variety of CHF prognostic prediction models have been established clinically [4, 5], of which the Seattle Heart Failure Model has the greatest impact. This model incorporates multiple risk factors and can accurately predict the survival rate up to 3 years and has received prospective verification [6]. However, owing to the inclusion of complex or unconventional clinical indicators, many models have not been fully utilized [7]. Most models predict survival time less than 3 years and lack of attention to the long-term prognosis of patients with CHF. More importantly, Seattle Heart Failure Model also faces the dilemma of failure in long-term prediction [6].

Herein, a 5-year survival prediction model of CHF patients induced by coronary heart disease (CHD) was developed to compensate for the clinical demand for long-lasting prediction. Previous studies have shown that traditional Chinese medicine (TCM) has accumulated rich experience in the treatment of CHF because of its significant increase in patients' quality of life and decrement in readmission rate [4]. However, the TCM intervention for CHF still lacks strong evidence of outcome indicators, such as mortality [8]. In this study, to simultaneously explore the effect of TCM on the long-term prognosis of patients with CHF, a 5-year survival prediction model was developed. First, the Hospital Information System (HIS) was used to extract patient data, and the Epidate data management software was used to unify and standardize the data. Then, the least absolute shrinkage selection operator Lasso regression was applied to filter the variables, and logistic regression was used to construct the model. Third, we constructed a nomogram, calculated the C-indices, and drew the ROC curve, calibration curve, and decision curve to evaluate the model performance. Finally, internal and external verification of the model was carried out. This work not only guides doctors for the prevention and treatment of CHF patients induced by CHD but also provides evidence for TCM intervention in CHF patients.

## 2. Materials and Methods

### 2.1. Study Design

#### 2.1.1. Participants

Seven Grade III Class A Chinese Medicine Hospitals in Shandong Province were surveyed. These were the Shandong University of TCM Affiliated Hospital, Weifang Chinese Medicine Hospital, Rizhao Chinese Medicine Hospital, Jinan Chinese Medicine Hospital, Qingdao Chinese Medicine Hospital, Zibo Chinese Medicine Hospital, and Weihai Chinese Medicine Hospital. Inpatients from January 1, 2012, to December 31, 2017, were investigated. The HIS was used to extract information on patients with CHF according to the International Disease Classification ICD-10 code.

#### 2.1.2. Inclusion Criteria

The inclusion criteria were as follows: (1) consistent with the Chinese Medical Association Cardiovascular Branch “Chinese Heart Failure Diagnosis and Treatment Guidelines” 2014 CHF diagnosis; (2) age between 45 and 75 years; (3) New York Heart Association functional classification (NYHA classification) II, III, IV; (4) CHF induced by CHD; (5) ejection fraction (EF) ＜50%; (6) follow-up time ≥5 years.

#### 2.1.3. Exclusion Criteria

The exclusion criteria were as follows: (1) acute HF; (2) patients who underwent coronary artery bypass surgery or cardiac resynchronization therapy; (3) associated with noncardiovascular events, such as malignant tumours and psychosis; (4) severe liver and kidney dysfunction; (5) missing data.

#### 2.1.4. Predictors

The predictors of the participants were all from the HIS, including 47 factors in four aspects:

1. Characteristics: age, sex, weight, days in the hospital, and course of CHF.
2. Comorbid diseases: hypertension, diabetes, hyperlipidemia, arrhythmia, cerebrovascular disease, respiratory disease, digestive system disease, kidney disease, peripheral vascular disease, and thyroid disease.
3. Treatment: cedilanid, digoxin, diuretics, spironolactone, nitrates, ACE inhibitor/angiotensin II receptor antagonist (ACEI/ARB), beta-blockers, aspirin, clopidogrel, anticoagulant drugs, calcium antagonists, trimetazidine, statins, and TCM intervention (TCM injection during hospitalization lasting no less than 10 days and Chinese patent medicine or Chinese medicine decoction lasting no less than 3 months during follow-up).
4. Physiological function and laboratory indices were as follows: heart rate, systolic blood pressure, diastolic blood pressure, NYHA classification, EF, left ventricular end-diastolic diameter (LVEDD), N-terminal probrain natriuretic peptide (NT-proBNP), serum potassium (K^+^), sodium (Na^+^), creatinine (CR), alanine transferase (ALT), total bilirubin (TBIL), triglyceride (TG), high-density lipoprotein (HDL-C), low-density lipoprotein (LDL-C), blood glucose (GLU), activated partial prothrombin time (APTT), and hemoglobin (HB).

#### 2.1.5. Data Extraction Basic and Management

HIS was used to obtain information on patients with CHF who satisfied the inclusion criteria in seven hospitals. Baseline characteristics, comorbid diseases, routine western medicine treatment, TCM intervention, physiological function indices, and laboratory indices of patients were recorded during hospitalization. Data were entered into the Epidate 3.1 data management software and integrated with Excel 2019 software.

#### 2.1.6. Follow-Up

Telephones were used as the main approach for the follow-up. Clinical follow-up was also applied as a supplementary. The follow-up ended on December 31, 2019, or until the patient died. Lost follow-up refers to those patients out of contact because of the patient's uncooperativeness, changing the mobile phone number, phone downtime, or not answering the phone call three or more times. According to the methodological requirements and to improve the credibility of the research results, the loss of follow-up rate was set to less than 15%. Several measures to reduce the loss to follow-up rate and improve the authenticity of follow-up are as follows. (1) Choice of follow-up time: avoiding the patient's rest time. (2) Training of follow-up researchers, providing language training to improve the communication skills of researchers; conducting professional knowledge training so that follow-up researchers can answer patients' consultations about their diseases and provide relevant suggestions. (3) Preparation for follow-up: the standard follow-up questionnaires should be prepared in advance. Researchers should review the cases, familiarize themselves with each patient's condition in advance, and set up a standardized questioning mode according to the patient's condition.

#### 2.1.7. Statistical Analysis

The random number table was used to randomly divide the seven hospitals into two groups (training set and verification set). Categorical variables were classified according to clinical standards. Continuous variables were converted into categorical variables according to clinical routine critical points. *R* 3.6.3 software was used for model construction and verification. The Lasso method was used to filter the best predictors from 47 features for its ability in high-dimensional data compression [9, 10]. Multivariate logistic regression analysis was used to construct the prediction model. After calculating the 95% confidence interval (95% CI), odds ratio, and *P* value of independent predictors, a nomogram was drawn to visualize the results of the predicted model. The C-indices were used to evaluate the discriminative ability of the model, and a receiver operating characteristic (ROC) curve was plotted to visualize the area under the curve (AUC) value. A calibration curve was applied to visualize the results of Hosmer–Lemeshow and calibrate this model. The clinical decision curve analysis (DCA) evaluates the clinical utility of the model [11, 12]. 1000 bootstrap resamples were used for internal verification, and the verification set data were used for external verification. A *P* value of less than 0.05 was considered statistically significant.

## 3. Results

### 3.1. Participants

**Figure 1:**
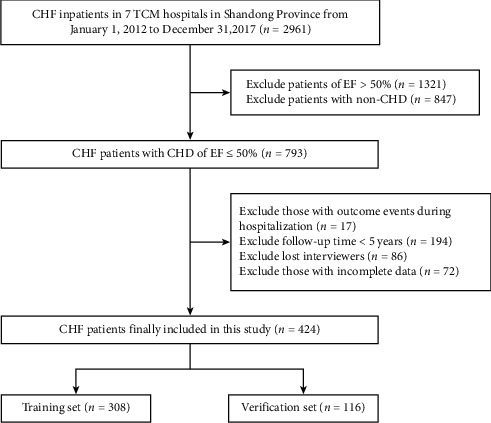
The flow chart of participant enrollment.

The study included 2961 patients with CHF from seven hospitals. Among them, there were 847 patients whose inducements were not CHD. Additionally, 1321 patients with EF ≥ 50% were excluded. Therefore, a total of 793 patients with CHF induced by CHD with EF ＜50% were selected. 17 patients had serious outcome events (i.e., cardiogenic death, worsening of HF, cardiogenic shock, and other reasons for death) during hospitalization, 194 patients were followed up for less than 5 years, 86 patients lost contact, and 72 patients had incomplete data. Finally, a total of 424 participants were qualified from seven hospitals that were randomly divided into two groups. Five hospitals were assigned to the training set (*n* = 308), and the remaining two hospitals were assigned to the verification set (*n* = 116). The flow chart of participant enrollment is shown in Figure 1.

### 3.2. Characteristics of Patients in the Training Set and Verification Set

Table 1 lists the characteristics of the patients in both the training and verification sets. After a 5-year follow-up, 215 (50.71%) patients died, and 209 (49.29%) patients survived. The median follow-up time of all patients was 61.4 months (interquartile [IQR], 18.13-71.59 months); the median follow-up time of the dead patients was 18.94 months (IQR, 9.07−35.87 months), and the median follow-up time of the survived patients was 71.6 months (IQR, 68.48−81.68 months). The median age was 70 years (IQR, 60−75 years) with a sex difference of 270 (63.68%) males and 154 (37.20%) females. In addition, 228 cases (53.77%) were treated with TCM, and 196 cases (46.23%) were not.

### 3.3. Variable Screening

**Figure 2:**
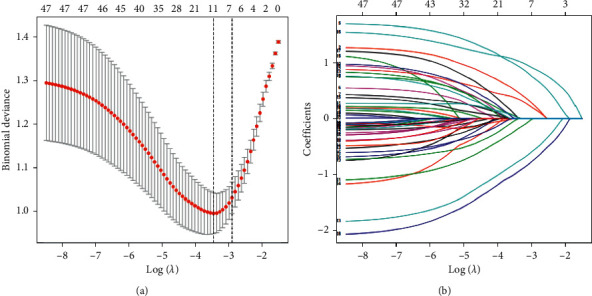
Lasso regression to screen variables. (a) Minimum criterion of the five-fold cross-validation Lasso model for optimal variables screening plotted with the likelihood deviation (binomial deviation) curve and the log (*λ*) curve. The minimum standard and the minimum standard of 1se (1se standard) were drawn as dotted vertical lines at the optimal value. (b) The Lasso regression shrinkage coefficient map of 47 features, according to the log (*λ*) sequence, draws the coefficient profile.

After screening with Lasso regression, 11 potential predictors, as shown in Figure 2 (approximately 4 : 1 ratio), were left for further prediction. The remaining variables included age, course of CHF, ACEI/ARB, aspirin, TCM intervention, NYHA classification, EF, NT-proBNP, K, TBIL, and GLU.

### 3.4. Logistic Regression

**Figure 3:**
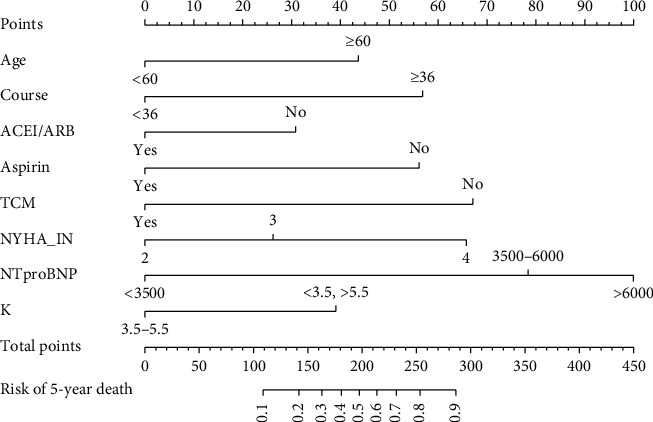
Five-year survival prediction nomogram of CHF patients induced by CHD.

Table 2 shows the results of the logistic regression. We determine whether it is an influencing factor based on the *P* value and then determine if it is a protective or injury factor by the negative or positive value of the “*β”.* Age, course of CHF, ACEI/ARB, aspirin, TCM intervention, NYHA, NT-proBNP, and K^+^ were independent predictors of 5-year mortality. Age, course of CHF, NYHA, NT-proBNP, K^+^ were injury factors, and ACEI/ARB, aspirin, TCM intervention were protective factors.  Figure 3 shows the nomogram of the model.

Nomogram: age, course of CHF, ACEI/ARB, aspirin, TCM intervention, NYHA classification, NT-proBNP, and K were used to obtain the 5-year mortality as potent covariates in CHF patients induced by CHD. For example, locating the age of patients by a vertical line up to the “points” axis was able to determine the age-related score. The score was 43 points for ages over 60. The total score was obtained by summing the scores of each variable using a similar process. Finally, positioning the total score on the “total points” axis, a vertical line up to the “Risk of 5-year death” axis represents the possibility of 5-year death for patients with CHF.

### 3.5. Model Verification

**Figure 4:**
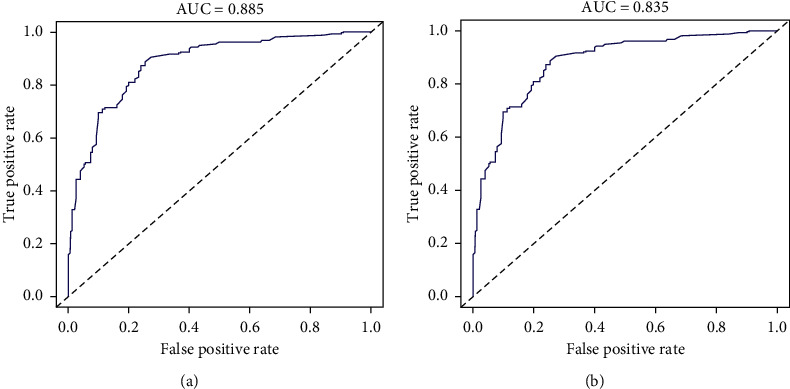
(a) ROC curve of the training set and (b) validation set.

**Figure 5:**
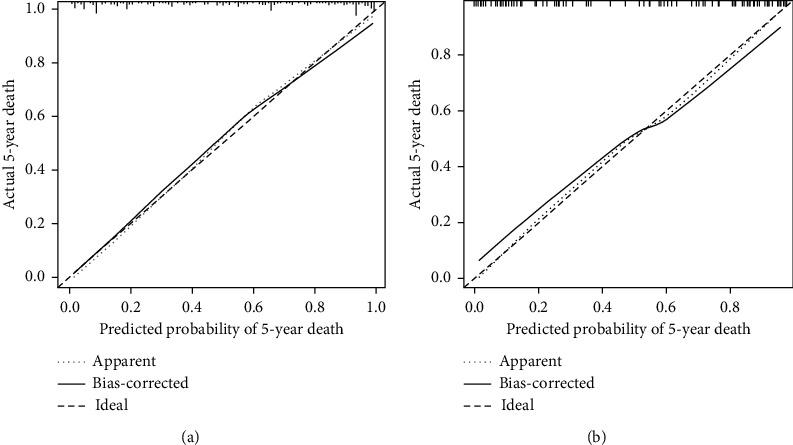
(a) Calibration curve of the training set and (b) validation set.

C-indices for the training set, bootstrap resamples, and verification set were 0.885, 0.867, and 0.835, respectively, indicating that the model has a good discriminating ability. The ROC curves of the training and validation sets are shown in Figure 4. Meanwhile, the DCA suggests a good agreement between the training and verification sets in Figure 5.

The diagonal line indicates that the area under the curve was 0.5, and the model did not work at this time. Thus, the larger the area under the blue curve, the larger the AUC value and the stronger the identification ability of the model.

The *X*-axis represents the predicted 5-year death, and the *Y*-axis represents the actual observed 5-year death. The diagonal dotted line represents a perfect prediction of the ideal model. The solid line indicates the performance of the prediction model, while the dashed line closer to the diagonal line indicates the corrected better prediction. Thus, the closer the solid line to the diagonal dotted line is, the closer the model prediction result to the actual one is.

### 3.6. Clinical Application

**Figure 6:**
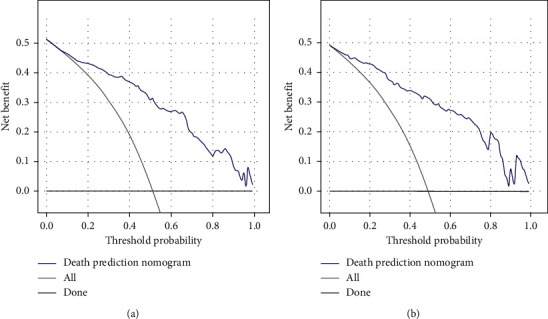
(a) DCA of the training set and (b) validation set.

According to the DCA of the 5-year mortality prediction model, when the threshold probability of the training set is in the range of 1–51%, and the threshold probability of the verification set is from 1% to 49%, and increased net benefit can be obtained by using this nomogram to predict death. As the risk of death increases within this threshold, the net benefit gradually decreases, indicating intervention implementation as early as possible. As shown in Figure 6, when the patient's 5-year mortality rate is predicted to be 50%, interventions in patients in advance may have a net benefit of approximately 30%.

Thick black line: suppose no one intervenes; thin black line, suppose everyone intervenes; blue line: actual intervention status.

## 4. Discussion

### 4.1. Main Findings and Significance

In this study, we first investigated TCM intervention as a factor and successfully developed a 5-year survival prediction model for patients with CHF induced by CHD. Eight independent predictors, age, course of CHF, ACEI/ARB, aspirin, TCM intervention, NYHA classification, NT-proBNP, and K, were confirmed when building this model. A previous study in the United States showed that as age increased, the mortality rate of CHF increased. Specifically, the one-year mortality rates of patients aged set 65−74, 75−84, and ≥85 years were 22%, 30.3%, and 42.7%, respectively, revealing a growing trend [13]. According to the results of our study, the 5-year mortality rate of CHF increased with disease progression, consistent with the progressive process of CHF exacerbation. Furthermore, a network meta-analysis study showed that sole ACEI would reduce the rate of all-cause death by 16%, while the decrease in the number of patients treated with ARB alone was 12%. Meanwhile, the effect of drug combination therapy (with *β* receptor blocker, corticosteroid receptor antagonist, and angiotensin receptor Nepal lysine inhibitor) was better than that of single-drug treatment [14]. Taking aspirin indefinitely is currently the standard treatment for CHD [15]. Although the detailed mechanism of action of aspirin is lacking, it can still reduce the major cardiovascular events and deaths of diagnosed CHD patients. Therefore, aspirin is generally recommended for patients with CHF induced by CHD [16].

The NYHA classification is a predictor of readmission and death of patients with CHF. A study by the European Society of Cardiology in Poland showed that the NYHA classification was an independent predictor of one-year death in patients hospitalized for CHF [17]. A multicenter cohort study with an average follow-up time of 6 years also showed that the NYHA classification is a risk factor for all-cause death [18]. The NT-proBNP level is an independent predictor of CHF death [19]. According to the guidelines, the prognosis of patients with CHF can be predicted by NT-proBNP, NYHA classification, and comorbidities. The addition of NT-proBNP has a better differentiation value for the prognosis of patients with CHF [20]. Studies have shown that low K and K levels are associated with increased mortality [21, 22]. Another study has shown that long-term monitoring of blood potassium levels is independently associated with death in patients with CHF, and persistent abnormal blood potassium is related to higher mortality [23]. The predictors included in this model once again proved previous research, indicating that the factors included in this model were reliable.

TCM is a historical tradition with over 2000-year development and has evolved unique theories and technical methods. It is featured in Chinese herbs and plays a key role in the treatment of malignant tumours, chronic diseases, and acute infectious diseases. In particular, the treatment of COVID-19 has played an important role worldwide. TCM has accumulated rich experience in the treatment of CHF because of its significant increase in myocardial contractility, cardiac hemodynamics, and patients' quality of life, leading to reduced readmission rate and mortality and improved long-term prognosis [4]. Moreover, TCM has good safety in clinical applications with rare side effects [24]. However, due to the difference in theories between TCM and western medicine, features in TCM treatment, such as complex intervention, multichannel, and multitarget, make TCM lack high-quality evidence. Therefore, it becomes a bottleneck for TCM to go abroad. In this study, multifactor regression analysis showed that TCM was a powerful protective factor against 5-year death in patients with CHF induced by CHD. For the first time, the TCM intervention factor is included in the prognostic model of patients with CHF, which provides reliable evidence for TCM intervention on CHF.

The use of predictive models in patients with CHF has become a standard for routine clinical practice. It helps to better identify high-risk groups that require more rigorous treatment and guide the rational allocation of medical resources. The nomogram is commonly used in cancer and other diseases because it can be applied to individual patients and consider several clinical variables to diagnose a disease or predict prognosis. In recent years, a large number of HF prediction models have been established to provide good support for the prevention and treatment of HF [25, 26]. For example, our nomogram assigned a 5-year death probability of 50% to a 60-year-old who had a 3-year HF course with NYHA III, NT-proBNP 3000 pg/ml, K^+^ 5.0, and who received conventional Western treatment but did not apply TCM. Using the model, we not only choose the predictors that have a greater impact on the 5-year mortality of patients with CHF induced by CHD but also automatically assigned quantitative weights to the predictors. The intensity of each factor's effect on the 5-year death was then determined to formulate individualized interventions. During verification, the constructed model showed good performance in terms of identification ability, calibration degree, and clinical benefit. Thus, this 5-year survival prediction model can not only guide doctors for the prevention and treatment of CHD patients induced by CHD but also provide evidence for TCM intervention for patients with CHF.

### 4.2. Advantages and Insufficiency

This study offers the following advantages. (1) HIS is used to extract clinical data, which is more complete and reliable, providing good data support for the real-world study. (2) most CHF prediction models paid more attention to the survival status for 1−3 years, while this study carried out a 5-year follow-up, which provides a direction for improving the treatment strategy of CHF patients with a more long-term prognosis. (3) TCM intervention factors were taken into consideration and successfully proved. Strikingly, a protective effect on the 5-year survival of CHF was demonstrated by TCM, indicating a reliable intervention method for treating CHF. (4) unlike previous models in CHF prediction, one of the most common causes, CHD, was applied as an impetus when conducting this 5-year prediction model. Meanwhile, we considered the possible impact of different EF on the outcome; patients with ejection fraction (HFrEF) and midrange ejection fraction (HFpEF) were selected. (5) The existing CHF prediction models cannot be fully used because they are complicated or contain unconventional clinical indicators. Therefore, this model contains eight factors to achieve a good prediction effect, namely, objective, sensitive, economical, and easily accessible.

However, there is some insufficiency to be further explored. (1) We excluded those patients whose data were missed, which may lead to selection bias. (2) To improve the accuracy of the model, this study restricts many conditions when screening the participants, resulting in a limited sample size, which may affect the accuracy of the results. (3) The long follow-up time of this study may cause lost to follow-up bias. (4) Due to the wide variety of TCM, we summed Chinese medicine injection, proprietary Chinese medicine, and Chinese medicine decoction as a comprehensive factor, which may affect the accuracy of the results. (5) Only eight out of 47 predictors were screened in this study based on Lasso regression in which the other 39 factors may be multicollinearity factors on the prognosis of HF.

## 5. Conclusion

In summary, the model was verified and proved to exhibit good performance. This study clarifies the 5-year prognostic factors of patients with CHF induced by CHD, provides evidence for TCM intervention on CHF under real-world research, and is likely to predict 5-year mortality. Because this study used retrospective data, the current electronic medical records of TCM have problems, such as confusing and complicated symptom terminology, untrue and irregular tongue and pulse diagnosis information, and a large amount of missing data. In addition, the problem of unstructured information extraction in electronic medical records has not yet been addressed. TCM syndrome research still lacks the support of clinical “real data,” so there are fewer TCM syndrome factors included in this study. To solve this problem, our research team explored an objective collection method for TCM syndromes. Furthermore, we will use the mature four-diagnosis instrument to collect TCM syndrome information, hoping to provide high-quality TCM syndrome data to construct models with TCM characteristics and then constantly improve and update the model.

## Figures and Tables

**Table 1:** Characteristics of patients in the training set and verification set after 5-year follow-up.

Variable	Training *n* (%)	Verification *n* (%)	Variable	Training *n* (%)	Verification *n* (%)
	(*n* = 308)	(*n* = 116)		(*n* = 308)	(*n* = 116)
5-year death			Clopidogre		
Yes	158 (51.30)	57 (49.14)	Yes	51 (16.56)	8 (6.90)
No	150 (48.70)	59 (50.86)	No	257 (83.44)	108 (93.10)
Basic characteristics			Anticoagulant		
Sex			Yes	121 (39.29)	27 (23.28)
Male	195 (63.31)	75 (64.66)	No	187 (60.71)	89 (76.72)
Female	113 (36.69)	41 (35.34)	Calcium antagonists		
Age (years)			Yes	59 (19.16)	22 (18.97)
＜60	78 (25.32)	20 (17.24)	No	249 (80.84)	94 (81.03)
≥60	230 (74.68)	96 (82.76)	Trimetazidine		
Weight (kg)			Yes	63 (20.45)	9 (7.76)
＜65	92 (29.87)	33 (28.45)	No	245 (79.55)	107 (92.24)
≥65	216 (70.13)	83 (71.55)	Traditional Chinese medicine		
Days in hospital			Yes	147 (47.73)	81 (69.83)
＜14	161 (52.27)	72 (62.07)	No	161 (52.27)	35 (30.17)
≥14	147 (47.73)	44 (37.93)	Physiological function and laboratory index		
Course (months)			Heart rate		
＜36	203 (65.91)	60 (51.72)	＜100	254 (82.47)	88 (75.86)
≥36	105 (34.09)	56 (48.28)	≥100	54 (17.53)	28 (24.14)
Comorbid disease			Systolic pressure		
Hypertension			＜140	188 (61.04)	60 (51.72)
Yes	172 (55.84)	79 (68.10)	≥140	120 (38.96)	56 (48.28)
No	136 (44.16)	37 (31.90)	Diastolic pressure		
Diabetes			＜90	218 (70.78)	65 (56.03)
Yes	67 (21.75)	45 (38.79)	≥90	90 (29.22)	51 (43.97)
No	241 (78.25)	71 (61.21)	NYHA_IN hospital		
Hyperlipidemia			2	34 (11.04)	10 (8.62)
Yes	87 (28.25)	13 (11.21)	3	183 (59.42)	60 (51.72)
No	221 (71.75)	103 (88.79)	4	91 (29.55)	46 (39.66)
Arrhythmia			EF		
Yes	229 (74.35)	116 (100)	≤40	125 (40.58)	66 (56.90)
No	79 (25.65)	0 (0)	＞40	183 (59.42)	50 (43.10)
Cerebral vascular disease			LVEDD		
Yes	187 (60.71)	116 (100)	35–55	101 (32.79)	37 (31.90)
No	121 (39.29)	0 (0)	＜35, ＞55	207 (67.21)	79 (68.10)
Respiratory diseases			NT-proBNP		
Yes	239 (77.60)	116 (100)	＜3500	173 (56.17)	55 (47.41)
No	69 (22.40)	0 (0)	3500–6000	99 (32.14)	50 (43.10)
Digestive system diseases			＞6000	36 (11.69)	11 (9.48)
Yes	158 (51.30)	114 (98.28)	CK		
No	150 (48.70)	2 (1.72)	＜200	290 (94.16)	108 (93.10)
Kidney disease			≥200	18 (5.84)	8 (6.90)
Yes	146 (47.40)	116 (100)	K		
No	162 (52.60)	0 (0)	3.5-3.5	268 (87.01)	104 (89.66)
Peripheral vascular disease			＜3.5, ＞5.5	40 (12.99)	12 (10.34)
Yes	146 (47.40)	116 (100)	Na		
No	162 (52.60)	0 (0)	135–145	226 (73.38)	91 (78.45)
Thyroid disease			＜135, ＞145	82 (26.62)	25 (21.55)
Yes	132 (42.86)	115 (99.14)	CR		
No	176 (57.14)	1 (0.86)	45–100	237 (76.95)	85 (73.28)
Treatment			＜45, ＞100	71 (23.05)	31 (26.72)
Cedilanid			ALT		
Yes	55 (17.86)	7 (6.03)	＜40	233 (75.65)	81 (69.83)
No	253 (82.14)	109 (93.97)	≥40	75 (24.35)	35 (30.17)

Digoxin			TBIL		
Yes	161 (52.27)	53 (45.69)	＜20	173 (56.17)	57 (49.14)
No	147 (47.73)	63 (54.31)	≥20	135 (43.83)	59 (50.86)
Diuretic			TG		
Yes	243 (78.90)	97 (83.62)	＜1.7	200 (64.94)	50 (43.10)
No	65 (21.10)	19 (16.38)	≥1.7	108 (35.06)	66 (56.90)

Spironolactone			HDLC		
Yes	222 (72.08)	91 (78.45)	＜1.8	292 (94.81)	109 (93.97)
No	86 (27.92)	25 (21.55)	≥1.8	16 (5.19)	7 (6.03)

Nitrates			LDLC		
Yes	185 (60.06)	49 (42.24)	＜4.1	297 (96.43)	111 (95.69)
No	123 (39.94)	67 (57.76)	≥4.1	11 (3.57)	5 (4.31)

ACE inhibitors/angiotensin II receptor antagonist			GLU		
Yes	133 (43.18)	37 (31.90)	＜6.1	170 (55.19)	46 (39.66)
No	175 (56.82)	79 (68.10)	≥6.1	138 (44.81)	70 (60.34)

Beta-blockers			APTT		
Yes	176 (57.14)	57 (49.14)	28–45	228 (74.03)	101 (87.07)
No	132 (42.86)	59 (50.86)	＜28, ＞45	80 (25.97)	15 (12.93)

Aspirin			HB		
Yes	193 (62.66)	66 (56.90)	＜110	43 (13.96)	10 (8.62)
No	115 (37.34)	50 (43.10)	≥110	265 (86.04)	106 (91.38)

**Table 2:** Independent predictors of 5-year mortality in CHF patients induced by CHD.

Intercept and variable	Prediction model		
	*β*	Odds ratio (95% CI)	*P* value
Intercept	−1.086	0.337 (0.086–1.234）	0.108
Age	1.153	3.169 (1.456–7.178)	0.004
Course	1.5095	4.525 (2.250–9.446)	＜0.001
ACEI/ARB	−0.8146	0.443 (0.228–0.839)	0.014
Aspirin	−1.5098	0.221 (0.107–0.436)	＜0.001
TCM	−1.6703	0.188 (0.09–0.365)	＜0.001
NYHA	1.3506	3.860 (1.218–12.946)	0.024
NT-proBNP 3500–6000	1.9021	6.700 (3.297–14.277)	＜0.001
NT-proBNP ＞6000	2.2320	9.318 (3.018–34.226)	＜0.001
K	1.2003	3.321 (1.209–9.458)	0.022

Note: *β*, the regression coefficient. Course, course of CHF.

## Data Availability

The data used to support the findings of this study are available from the corresponding author upon request.
